# β‐D‐Glucan Testing in Candidemia: Determinants of Positivity and Association With Mortality

**DOI:** 10.1111/myc.70067

**Published:** 2025-05-12

**Authors:** Karl Oldberg, Jakob Stenmark, Helena Hammarström

**Affiliations:** ^1^ Department of Clinical Microbiology Infection Prevention and Control, Office for Medical Services Lund Sweden; ^2^ Section for Infection Medicine, Department of Clinical Sciences Lund Lund University Lund Sweden; ^3^ Region Västra Götaland, Sahlgrenska University Hospital Department of Clinical Microbiology Gothenburg Sweden; ^4^ Department of Infectious Diseases, Institute of Biomedicine Sahlgrenska Academy, University of Gothenburg Gothenburg Sweden; ^5^ Region Västra Götaland, Sahlgrenska University Hospital Department of Infectious Diseases Gothenburg Sweden

## Abstract

**Background:**

Serum 1,3‐β‐d‐glucan (BDG) tests are frequently used for diagnosing invasive candidiasis. However, BDG tests remain negative in many patients with candidemia, and factors influencing the probability for positive test results are poorly understood.

**Objectives:**

To study clinical and microbiological factors predictive of a positive BDG test, as well as the association of a positive BDG test with mortality in patients with candidemia.

**Methods:**

In a retrospective cohort of patients with candidemia, BDG was analysed by the Glucatell assay and the Wako Beta‐Glucan Test. Predisposing conditions, focus of infection and other variables were retrieved from medical charts and laboratory databases. Their association with a positive BDG test, and the association between positive BDG and death was tested in univariate analysis and multivariable logistic regression.

**Results:**

We included 134 patients with candidemia. Positive BDG and a non‐abdominal deep‐seated focus of infection (e.g., hematogenously disseminated infection and deep mediastinal/pleural candidiasis) were positively correlated in univariate and multivariable analyses [Wako adjusted odds ratio 9.11 (95% CI 1.66–172, *p* = 0.039), Glucatell adjOR 9.14 (95% CI 1.66–172, *p* = 0.039)]. Having a positive BDG test increased the risk for 90 days mortality after controlling for potential confounders, mainly age, septic shock, and ICU admission [Wako adjOR 4.73 (95% CI 1.71–14.7, *p* = 0.0043), Glucatell adjOR 3.59 (95% CI 1.33–10.6, *p* = 0.015)].

**Conclusions:**

In patients with candidemia, a positive BDG test is more common in the presence of a concomitant non‐abdominal deep‐seated infection. Patients with a positive BDG test have a higher 90‐day mortality.

## 1 Introduction

Culture based diagnosis for invasive candidiasis is limited by a low sensitivity [1], and international guidelines recommend the use of non‐culture‐based assays as complementary diagnostic tests for invasive candidiasis [2, 3, 4]. 1,3‐β‐d‐glucan (BDG) is a predominant component of the cell wall of most pathogenic fungi, and diagnostic assays that detect BDG in serum are widely used in patients at risk of invasive candidiasis. The diagnostic performance of different BDG assays has been assessed in multiple studies, including several meta‐analyses [5, 6, 7, 8, 9, 10], but the results are diverse due to differences in study design, frequency and timing of sampling, patient‐related factors, and type of BDG‐assay [11, 12, 13]. Despite this, BDG assays play a significant role in the management of critically ill patients at risk for invasive candidiasis [2]. Apart from being an important complementary diagnostic test, repeated BDG testing can also help to assess the prognosis of patients with invasive candidiasis [14].

There is a large interindividual variation in BDG levels [15, 16], and recent studies found that 12%–53% of patients with candidemia had negative BDG tests at the time of the positive blood culture [17, 18, 19, 20]. Studies have shown that BDG levels may be low early in the course of an infection, but will then often turn positive after repeated testing [15, 21]. However, persistently negative BDG results in patients with candidemia have also been reported [22]. It has been suggested that factors such as fungal burden and severity of disease [22] as well as *Candida* species [18, 19] may influence the probability of obtaining a positive BDG test in patients with candidemia, but knowledge is still lacking. Therefore, additional insights that may aid clinicians in interpreting BDG test results are of utmost importance.

Using two separate BDG assays, we investigated the presence of different clinical factors, as well as the overall mortality, in candidemic patients with and without a positive BDG test result. The aims of the study were to identify clinical factors that may predict a positive BDG test in patients with candidemia and to assess if a positive BDG result is associated with an increased mortality.

## 2 Materials and Methods

We performed a retrospective study during July 2015 to October 2021 at Sahlgrenska University Hospital in Gothenburg and Skåne University Hospital together with seven regional hospitals in Region Skåne, Sweden. A case–control study design was employed to assess the optimal cut‐off levels for the two BDG assays to diagnose candidemia. Based on these cut‐off levels, we then evaluated predictors for positive BDG results and the association of BDG level with mortality in the cohort with candidemia.

### 2.1 Patient Enrolment

Cases with candidemia and controls were identified through the Clinical microbiology databases in Region Skåne and Gothenburg. Eligible cases were individuals with: (1) one or more blood cultures with growth of *Candida* species or other ascomycetous yeasts, and (2) a bio banked serum sample analysed for BDG obtained within 2 days before and 7 days after the sample date of the positive blood culture. Exclusion criteria were microbiological analyses indicative of an invasive fungal infection other than candidemia, collected within 30 days before or after the sampling date of the serum sample.

One matched control for each case was randomly selected from individuals with (1) blood cultures without growth of fungi and (2) a serum sample for BDG analysis obtained and bio banked as described above for the cases. Matching criteria were: year of sampling, sex, type of nursing clinic, and patient age ± 5 years. If no fully matched control was eligible, the age criterion was extended to ± 20 years for adults, followed by dropping the sex criterion or type of nursing clinic if necessary. Microbiologic data was subsequently reviewed, and controls with any suspected invasive fungal infection were excluded from the study.

### 2.2 Data Collection and Definitions

Medical charts were reviewed to collect background information and clinical data regarding fungal infections and pre‐defined factors that were expected to possibly influence the BDG levels and mortality: Age, sex, type of nursing clinic, admission to an intensive care unit (ICU), comorbidities, performed surgery, haematopoietic stem‐cell transplantation, solid organ transplantation, septic shock, antifungal treatment, neutropenia, date of death, number and date of blood cultures, culture results, time to blood culture positivity, imaging results and results of ophthalmological examination. Patients were grouped according to the type of infection at the time of candidemia. ‘Intra‐abdominal candidiasis (IAC)’ was defined based on the recovery of a yeast in a sample collected peri‐operatively from the abdominal cavity or from a drain placed < 24 h before sampling [4]. Patients with recent abdominal surgery, who had growth of yeast from an abdominal drain placed > 24 h before culture sampling, were re‐evaluated by a senior infectious disease specialist (H.H.) and were classified as IAC if clinical and radiologic signs as well as treatment response was compatible with an intra‐abdominal infection. Deep‐seated infections, other than IAC, were classified as follows. ‘Hematogenous dissemination’ was defined as (1) growth of yeast in samples obtained by a sterile procedure from a normally sterile site other than the abdominal cavity, with radiological or clinical signs of disseminated infection or (2), small, target‐like abscesses in liver or spleen (bull's‐eye lesions) or in the brain, or, meningeal enhancement within 2 weeks from a positive blood culture [4]. ‘Deep mediastinal or pleural candidiasis’ was defined as growth of yeast in samples obtained by a sterile procedure from mediastinal or pleural fluid or tissue, within 2 weeks from the positive blood culture, in patients that had undergone thoracic surgery [4]. Proven infective endocarditis was defined according to the modified Duke criteria [23]. Pyelitis was defined based on a typical clinical picture and the recovery of *Candida* in a urine sample within a few days from the positive blood culture. The cases were defined as ‘Candidemia only’ if there was no evidence of a focal infection at the time of the recovery of *Candida* in blood culture.

### 2.3 Analysis of BDG With the Glucatell Assay

Fresh serum samples were analysed by the colorimetric Glucatell assay kit (Ass. Cape Cod, MA, USA) according to the manufacturer's instructions, modified to include an extra dilution step yielding an interval of quantification of 100 to 800 pg/mL. The Glucatell assay is the initial BDG assay developed by Ass. Cape Cod, MA, USA, which was later FDA‐approved and made available as a commercial diagnostic test under the name of the Fungitell assay. The Glucatell and the Fungitell assays are fundamentally identical, with the only distinction being the method of serum pre‐treatment (heat treatment at 75°C and pre‐treatment reagent based on KOH and KCl for Glucatell and Fungitell, respectively). To make truncated results compatible with calculations, samples with concentrations below the lower limit of quantification (< 100 pg/mL) or above the upper limit of quantification (> 800 pg/mL) were set to a concentration of 1 pg/mL below or above the limit in the data analysis (i.e., 99 pg/mL and 801 pg/mL, respectively).

### 2.4 Analysis of BDG With the Wako Beta‐Glucan Test

Analyses with the turbidimetric Beta‐glucan test (Wako assay) (FUJIFILM Wako Pure Chemical Corporation, Osaka, Japan) were performed on frozen aliquots of the serum samples previously analysed by the Glucatell assay. The time elapsed between the two analyses was between one and 6 years. Analyses were done according to the instructions of the manufacturer, using the MT‐6500 instrument. For concentrations above 600 pg/mL, the heat‐treated sample was diluted with sample diluent 1:10 and re‐analysed to reach a definite concentration. Concentrations below the lower limit of quantification (< 3.231 pg/mL) were set to the first value under the limit, i.e., 3.230 pg/mL.

### 2.5 Analysis for Possible Degradation of BDG in Frozen Serum Samples

To exclude that the concentration of BDG had decreased during storage at‐20°C, 21 serum samples with positive BDG results that had been analysed with Glucatell in 2015 were thawed and reanalyzed in 2020.

### 2.6 Statistical Analysis

Statistical analyses were performed using R version 4.2.3. The difference in concentrations in the analysis of possible BDG degradation was assessed by the paired t‐test. Diagnostic performance was analysed by receiver‐operating characteristics (ROC) curve analysis. The difference between the areas under the curve (AUC) of the two assays was compared using DeLong's test for two correlated ROC curves. Optimal cut‐offs were chosen based on the highest Youden index. Confidence intervals for sensitivity and specificity were calculated with the exact binomial test.

To evaluate the correlation between different variables and a positive BDG test, and to adjust for possible confounders, odds ratios in univariate and adjusted odds ratios in multivariable models were calculated with binary logistic regression. Separate models were constructed for each of the two BDG assays. Pre‐defined variables deemed to be relevant for the outcome were included: Age (dichotomized as under or ≥ 18 years), ICU admission, haematologic malignancy, neutropenia, abdominal surgery, other surgery, yeast species in blood culture, septic shock, type of infection (at first candidemia only vs. any deep infection, secondly subdivided into IAC or other deep‐seated infection vs. candidemia only), ongoing antifungals at BDG sampling, blood culture time to positivity (dichotomized at the median of 3 days), and time from confirmed invasive candidiasis to BDG sampling (dichotomized at the median of 3 days). Variables with *p* < 0.2 in univariate analysis for any of the two assays were incorporated in the multivariable analyses for both assays. However, due to probable collinearity between the variables ´Abdominal surgery´ and IAC, we decided to exclude ´Abdominal surgery´ from the model as it is clinically of less interest than IAC. The same methods were used for the analyses for a correlation between different variables and 30‐ and 90‐day mortality. Included variables were positive BDG test, age, sex, ICU admission, species in blood culture, haematologic malignancy, neutropenia, abdominal surgery, other surgery, septic shock, and type of infection. These multivariable models were only constructed to adjust for potential confounding in the correlation between a positive BDG test and 90‐day mortality, not to estimate the effects of the covariates. Non‐normally distributed variables are presented as medians with interquartile ranges. The threshold for statistical significance was set to *p* < 0.05.

### 2.7 Ethics Statement

The study was approved by the Swedish Ethical Review Authority (Dnr 2020–00243).

## 3 Results

### 3.1 Study Population

142 cases of yeast blood stream infection with concurrent serum samples for BDG‐analysis were identified. Of these, seven were excluded because of another proven or possible invasive fungal infection within 30 days (5 with invasive aspergillosis, 1 with *Pneumocystis jirovecii* pneumonia, 1 with invasive fusariosis) and one was excluded due to a missing serum sample. This left 134 cases. Out of 134 controls initially selected, two were excluded due to a possible invasive fungal infection, leaving a total of 132 controls in the study. Among the 132 controls, 120 were fully matched to the cases according to all criteria. In 7 controls, the age criterion was extended, and in 5 controls, the sex criterion or type of nursing clinic had to be dropped.

**FIGURE 1:**
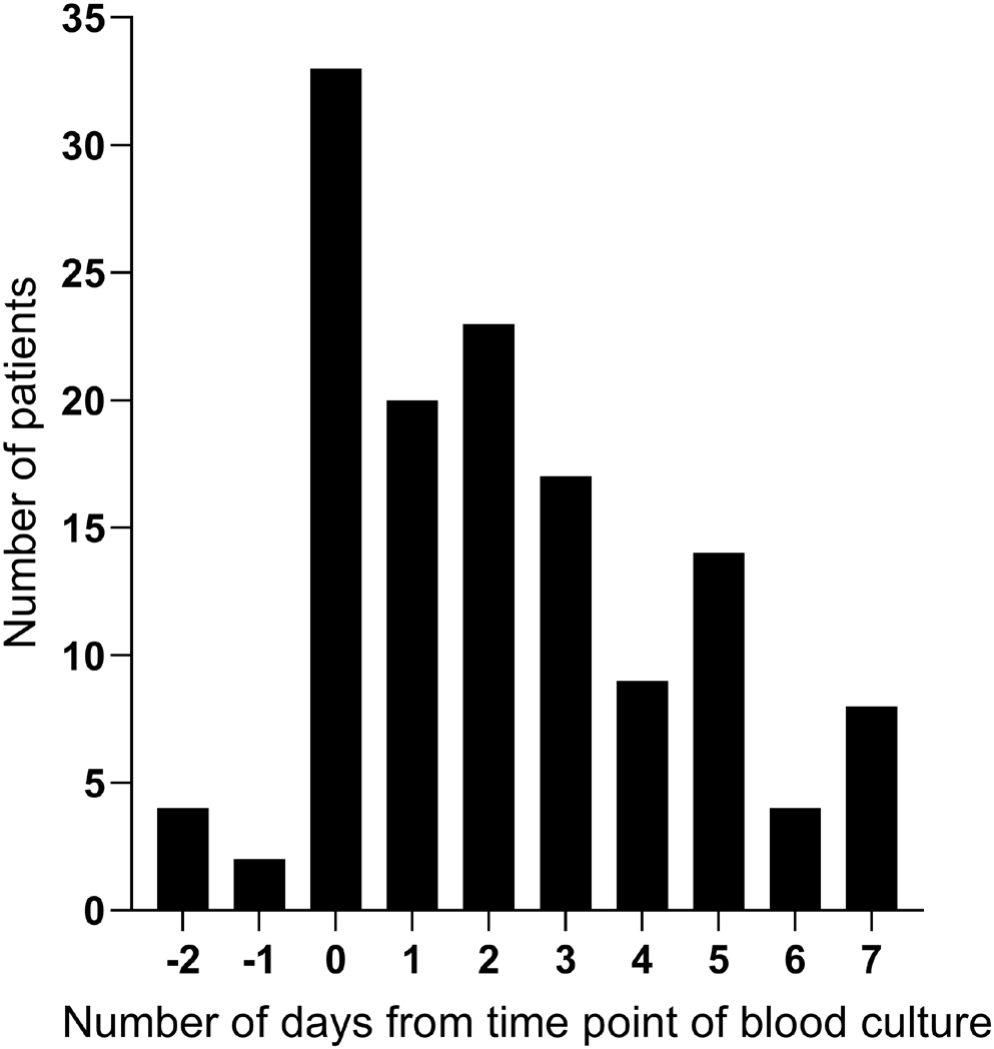
The timing of Beta‐D‐glucan sampling in relation to blood culture sampling among cases.

Background information of the cases and controls is presented in Table 1. *Candida albicans* was the most common species (49%), followed by *Candida glabrata* (15%) and *Candida parapsilosis* (9%). Among the patients with candidemia, 38 (28%) had a concomitant deep‐seated infection. IAC was the most common deep focus (21 patients, 16%), followed by hematogenous dissemination to various organs (*n* = 8) and mediastinal or pleural infection following thoracic surgery (*n* = 7). The timing of BDG‐sampling in relation to blood culture sampling is presented in Figure 1.

**TABLE 1:** Background data for the cases of candidemia and for controls.

	Cases (*n* = 134)	Controls (*n* = 132)
Age (median, IQR)	65 (48–71)	65 (48–71)
Male, *n* (%)	89 (66)	88 (67)
Predisposing risk condition^a^		
ICU admission^b^, *n* (%)	67 (50)	71 (54)
Haematological malignancy, *n* (%)	18 (13)	25 (19)
Allogenic HSCT, *n* (%)	4 (3)	14 (11)
Solid tumour, *n* (%)	25 (19)	26 (20)
Neutropenia^c^ within last 10 days, *n* (%)	24 (18)	
Solid organ transplant, *n* (%)	10 (8)	6 (5)
Abdominal surgery, *n* (%)	38 (28)	15 (11)
Other surgery, *n* (%)	21 (16)	19 (14)
Species, *n* (%)		
*Candida albicans*	66 (49)	
*Candida glabrata*	20 (15)	
*Candida parapsilosis*	12 (9)	
*Candida krusei* (*Pichia kudriavzevii*)	6 (4)	
*Candida tropicalis*	6 (4)	
*Candida dubliniensis*	6 (4)	
*Candida lusitaniae* (*Clavispora lusitaniae*)	5 (4)	
*Candida lambica* ( *Pichia fermentans* )	1 (0.7)	
*Candida pelliculosa* (*Wickerhamomyces anomalus*)	1 (0.7)	
*Candida utilis* (*Cyberlindnera jadinii*)	1 (0.7)	
*Candida kefyr* (*Kluyveromyces marxianus*)	1 (0.7)	
*Saccharomyces cerevisiae*	1 (0.7)	
*Saprochaete capitata*	1 (0.7)	
Mixed species^d^	7 (5)	
Types of Candida infection, *n* (%)		
Candidemia only	96 (72)	
Candidemia with deep‐seated infection	38 (28)	
Intraabdominal candidiasis	21 (16)	
Other deep‐seated infection	17 (13)	
Hematogenous dissemination^e^	8	
Deep mediastinal or pleural candidiasis after thoracic surgery	7	
Endocarditis	1	
Pyelitis	1	

Abbreviations: HSCT, haematopoietic stem cell transplantation; ICU, Intensive care unit; IQR, interquartile range.

^a^ Conditions not mutually exclusive.

^b^ Admitted to ICU on the day of or within 3 days prior to blood culture yielding Candida.

^c^ Neutrophil granulocytes < 500/mL. Data only collected for cases.

^d^ *C. albicans* and *C. glabrata* (*n* = 3), *C. albicans* and *C. parapsilosis* (*n* = 2), *C. tropicalis* and *C. parapsilosis* (*n* = 1), *C. glabrata* and *C. dubliniensis* (*n* = 1).

^e^ Dissemination to one or several of the following organs (liver, spleen, kidneys, skin, eyes and brain, *n* = 6), joint/septic arthritis (*n* = 1), soft tissue/muscle compartment (*n* = 1).

### 3.2 Analysis for Possible Degradation of BDG in Frozen Serum Samples

Twenty‐one frozen serum samples with initial BDG concentrations between 158 and 399 pg/mL were reanalyzed with the Glucatell assay after 5 years of storage. The mean change in BDG concentration was + 10 pg/mL (95% CI −16 to +36), with a mean coefficient of variation of 7% (i.e., within the normal variation of the analysis). One sample with an initial concentration of 158 pg/mL changed its classification from positive to negative (< 100 pg/mL) after reanalysis.

### 3.3 Diagnostic Performance and Optimal Cut‐Off Levels

**FIGURE 2:**
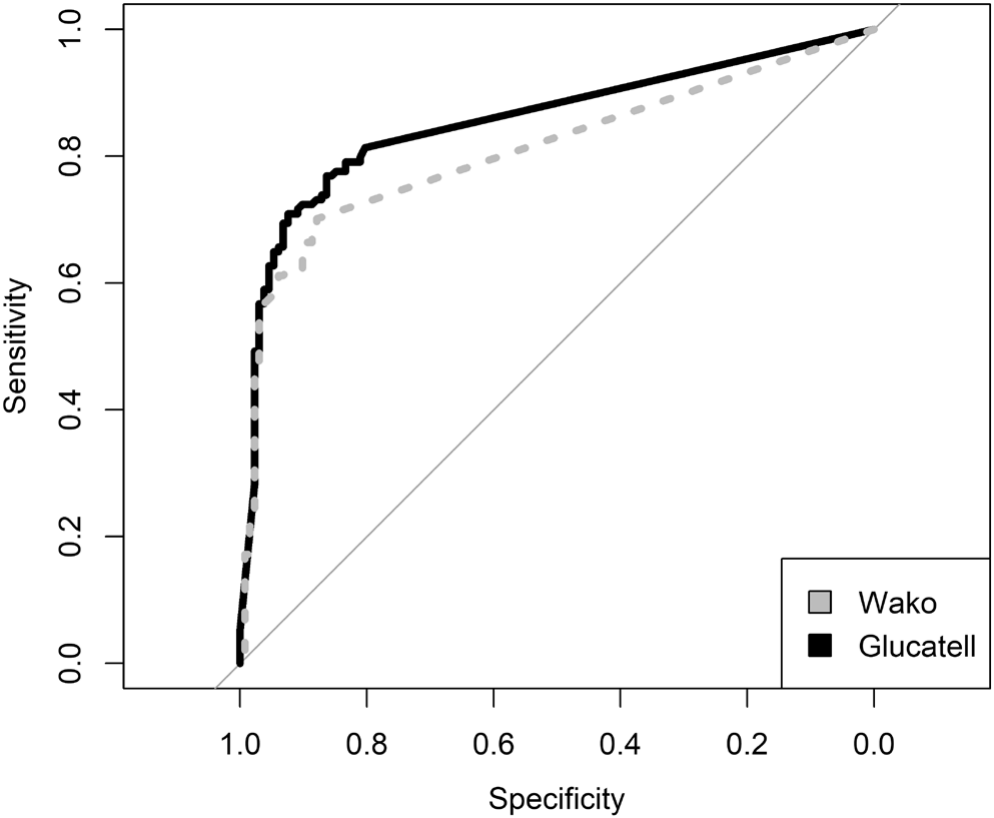
Receiver‐operating characteristics (ROC) plot of the beta‐D‐glucan test, with sensitivity and specificity for candidemia. The area under the ROC curve (AUC) of the Wako assay was 0.81 (95% confidence interval 0.76–0.86) and the AUC of the Glucatell assay was 0.86 (95% CI 0.81–0.90).

The ROC curve AUC was 0.81 (95% CI 0.76–0.86) for the Wako assay and 0.86 (95% CI 0.81–0.90) for the Glucatell assay, Figure 2. The difference between the two AUCs was statistically significant (*p* < 0.001). For Wako, at the cut‐off recommended by the manufacturer (7 pg/mL), the sensitivity was 0.59 (95% CI 0.50–0.67) and the specificity was 0.95 (95% CI 0.89–0.98). The cut‐off with the highest Youden index was ≥ 3.231 pg/mL, equal to the lowest detection threshold with the reagents used. At this cut‐off, the sensitivity was 0.70 (95% CI 0.62–0.78) and the specificity was 0.88 (95% CI 0.81–0.93). For Glucatell, there is no manufacturer‐recommended cut‐off for in vitro diagnostics. The cut‐off with the highest Youden index was ≥ 169 pg/mL, with a sensitivity of 0.71 (95% CI 0.62–0.78) and a specificity of 0.92 (95% CI 0.87–0.96). The cut‐offs 3.231 pg/mL for Wako and 169 pg/mL for Glucatell were used in subsequent analyses.

### 3.4 Variables Predictive of a Positive BDG Test in Patients With Candidemia

**FIGURE 3:**
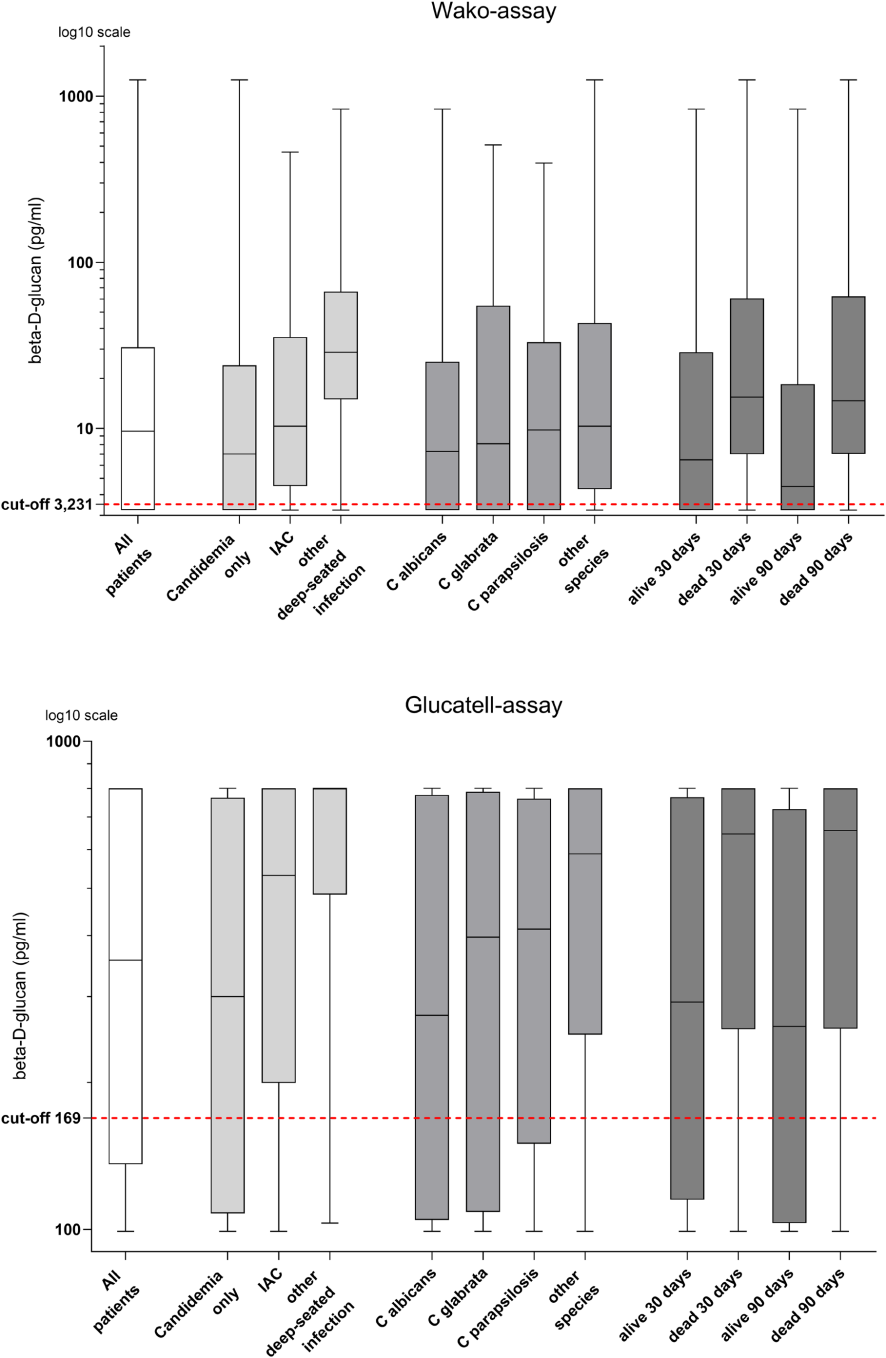
Distribution of beta‐D‐glucan concentrations measured by the Wako and the Glucatell assays. Beta‐D‐glucan (BDG)‐results below the lowest limit of detection were set to the first concentration under the detection limit (i.e., Wako < 3.231 pg/mL as 3.230 pg/mL and Glucatell < 100 pg/mL as 99 pg/mL). Glucatell BDG‐results above the highest limit of detection were similarly set to the first concentration above the detection limit (i.e., > 800 pg/mL as 801 pg/mL). Horizontal lines in the boxes represent the medians and whiskers represent minimum and maximum values. The y‐axis is set to log10 scale.

Using the calculated optimal cut‐off levels, 94 (70%) and 95 (71%) of the patients with candidemia had a positive BDG test with the Wako and Glucatell assay, respectively. Abdominal surgery and deep‐seated infection other than IAC were associated with a positive BDG test in univariate analysis (Table 2). In multivariable logistic regression analysis, incorporating covariates that had a *p*‐value under 0.2 in univariate analysis for any of the two assays (age, other yeast species, septic shock, IAC, and deep‐seated infection other than IAC), only deep‐seated infection other than IAC remained significantly associated with a positive BDG test [Wako adjOR 9.11 (95% CI 1.66–172), Glucatell adjOR 9.14 (95% CI 1.66–172)]. The distribution of BDG concentrations in all patients and in patients grouped according to *Candida* species, type of infection, and death are presented in Figure 3.

**TABLE 2:** Univariate and multivariable analyses of factors associated with positive beta‐D‐glucan test in patients with candidemia.

	Glucatell				Wako			
	Univariate		Multivariable		Univariate		Multivariable	
	OR (95% CI)	*p*	adjOR (95% CI)	*p*	OR (95% CI)	*p*	adjOR (95% CI)	*p*
Age < 18 years (*n* = 13)	0.438 (0.136–1.45)	0.16	0.401 (0.104–1.47)	0.17	0.456 (0.141–1.51)	0.18	0.399 (0.103–1.47)	0.16
ICU admission (*n* = 67)	0.805 (0.378–1.70)	0.57	—		0.867 (0.411–1.82)	0.71	—	
Haematologic malignancy (*n* = 18)	1.08 (0.374–3.57)	0.89	—		1.12 (0.390–3.72)	0.84	—	
Neutropenia^a^ (*n* = 24)	1.01 (0.394–2.82)	0.98	—		1.05 (0.412–2.94)	0.91	—	
Abdominal surgery (*n* = 38)	2.79 (1.12–8.01)	0.038	—		2.32 (0.964–6.24)	0.074	—	
Other surgery (*n* = 21)	1.03 (0.382–3.10)	0.95	—		0.825 (0.313–2.35)	0.70	—	
Species in blood culture^b^								
*C. albicans* (*n* = 66)	0.665 (0.306–1.42)	0.29	—		0.622 (0.287–1.32)	0.22	—	
*C. glabrata* (*n* = 20)	0.792 (0.295–2.28)	0.65	—		0.828 (0.309–2.38)	0.71	—	
*C. parapsilosis* (*n* = 12)	0.875 (0.257–3.45)	0.84	—		0.911 (0.268–3.59)	0.89	—	
Other species (*n* = 29)	2.55 (0.955–8.10)	0.080	2.22 (0.788–7.32)	0.15	2.67 (1.00–8.47)	0.067	2.32 (0.825–7.62)	0.13
Septic shock (*n* = 23)	2.19 (0.754–7.97)	0.18	1.78 (0.553–6.88)	0.36	2.28 (0.787–8.30)	0.16	1.99 (0.624–7.71)	0.27
Type of infection^c^								
Deep seated infection (*n* = 38)	3.62 (1.39–11.3)	0.014			2.92 (1.18–8.38)	0.030		
IAC (*n* = 21)	2.33 (0.787–8.59)	0.16	1.84 (0.574–7.12)	0.33	1.75 (0.625–5.74)	0.31	1.33 (0.433–4.62)	0.63
Other deep‐seated infection (*n* = 17)	8.77 (1.68–162)	0.039	9.14 (1.66–172)	0.039	8.77 (1.68–162)	0.039	9.11 (1.66–172)	0.039
Ongoing antifungals^d^ (*n* = 55)	1.16 (0.546–2.53)	0.70	—		1.44 (0.674–3.15)	0.35	—	
Time to positivity of blood culture^a^ < 3 days (*n* = 69)	0.959 (0.449–2.04)	0.91	—		1.03 (0.488–2.19)	0.93	—	
Time from confirmed IC to BDG sample < 3 days (*n* = 79)	0.859 (0.395–1.83)	0.70	—		0.940 (0.437–1.99)	0.87	—	

Abbreviations: adjOR, adjusted odds ratio; IAC, Intra‐abdominal candidiasis; IC, invasive candidiasis; ICU, intensive care unit.

^a^ One case with missing data on neutropenia and one case with missing data on time to positive culture were excluded in univariate analyses.

^b^ Seven cases with multiple yeast species were excluded from univariate analysis of species in blood culture and in the multivariable analysis.

^c^ IAC and other deep seatedinfectionsn are subgroups. Both subgroups were included in multivariable analysis, candidemia only is the reference category.

^d^ At the day of BDG sampling.

### 3.5 Mortality

The overall mortality was 35% at 30 days and 43% at 90 days. The odds ratios for death at 30 days were 2.34 (95% CI 1.03–5.72) in patients with a positive Wako BDG test and 2.22 (95% CI 0.978–5.44) in patients with a positive Glucatell BDG test. At 90 days, the odds ratios for death increased to 3.75 (95% CI 1.66–9.16) when having a positive Wako BDG test and 2.96 (95% CI 1.33–7.02) when having a positive Glucatell BDG test. To adjust for potential confounding, a logistic regression model was constructed including covariates with a *p*‐value < 0.2 in univariate analysis for any of the two assays (age, ICU admission, haematological malignancy, non‐abdominal surgery, septic shock, and in the models for 90‐days mortality, IAC and other deep‐seated infection in addition) (Tables S1–S4). The adjusted odds ratios for death at 30 days were 2.35 (95% CI 0.942–6.27) in patients with a positive Wako assay, and 2.25 (95% CI 0.898–6.06) in patients with a positive Glucatell assay. For the 90 days mortality, the adjusted odds ratio for patients with a positive Wako test was 4.73 (95% CI 1.71–14.7), and 3.59 (95% CI 1.33–10.6) in patients with a positive Glucatell test (Table 3).

**TABLE 3:** Mortality at 90 days, with positive beta‐D‐glucan test as exposure, in patients with candidemia.

	Glucatell				Wako			
	Alive 90 days, *n* = 76	Dead 90 days, *n* = 58	Crude OR 95% CI	Adjusted^a^ OR 95% CI	Alive 90 days, *n* = 76	Dead 90 days, *n* = 58	Crude OR 95% CI	Adjusted^a^ OR 95% CI
Positive BDG test	47 (62%)	48 (83%)	2.96 (1.33–7.02)	3.59 (1.33–10.6)	45 (59%)	49 (84%)	3.75 (1.66–9.16)	4.73 (1.71–14.7)

^a^ The adjusted OR were calculated in a multivariable logistic regression model including the variables that had *p* < 0.2 in univariate analyses: Age, ICU admission, haematological malignancy, non‐abdominal surgery, septic shock and deep‐seated infection (Tables S3 and S4).

## 4 Discussion

This study shows that in patients with candidemia, a positive BDG test was associated with a deep‐seated infection other than IAC (i.e., hematogenously disseminated infection and deep mediastinal or pleural candidiasis) and with a higher probability of death at 90 days. These results were consistent for both the Wako and the Glucatell assays, indicating that the results are not dependent on a specific method for measuring BDG.

The Wako assay had a slightly lower ROC curve AUC than the Glucatell assay, meaning a somewhat lower ability to discriminate patients with invasive yeast infection from negative controls. These results are in line with the results of previous studies comparing Wako with the Fungitell assay (i.e., the commercial version of the Glucatell assay) in the diagnosis of candidemia, where the Wako test showed a lower sensitivity [12]. In the present study, the optimal cut‐off levels were 3.231 pg/mL for the Wako assay (lower than that recommended by the manufacturer) and 169 pg/mL for the Glucatell assay (higher than the 80 pg/mL cut‐off recommended for Fungitell). Even with these cut‐off levels, approximately 30% of patients with candidemia did not test positive for BDG. Although only a few previous studies have reported a sensitivity for BDG higher than 80% in diagnosing invasive candidiasis, international guidelines recommend the use of a BDG assay as a complementary diagnostic test for these patients [2, 3, 4]. Gaining a deeper insight into how to interpret BDG results is therefore of utmost importance.

We found that candidemic patients with a deep focus of infection other than IAC, such as patients with hematogenously disseminated infection and deep mediastinal or pleural candidiasis, were more likely to have a positive BDG test. This may be explained by a higher fungal load in patients with disseminated infection compared to patients with candidemia and no deep focal infection, leading to an increased serum BDG concentration. Consistent with this, Agnelli et al. showed that repeatedly negative BDG results were more frequently encountered in patients who had growth of *Candida* in only one set of blood cultures and in patients with less severe clinical presentations, indicating a correlation between BDG level and fungal burden in blood [22]. The kinetics of BDG may also be involved. In deep‐seated candidiasis, the candidemic episode may be secondary to an already established infectious focus, and BDG may then leak into the circulation and reach high concentrations before blood cultures turn positive. This was also discussed in a paper by Träger et al. where patients with a deep‐seated *Candida* infection were more likely to have a positive BDG test prior to the positive blood culture compared to patients with a more rapid onset candidemia such as that derived from a colonised central venous catheter [20]. Surprisingly, we found no statistically significant correlation between a positive BDG test and a deep‐seated infection in the abdominal cavity (IAC) in patients with candidemia. There was a trend towards an association, but since the effect size was smaller than that of other deep‐seated infections, the sample size may have been insufficient to prove an association. In a clinical context, our findings suggest that in patients with candidemia, elevated BDG concentrations may warrant further investigation for potential deep‐seated foci of infection. Moreover, our results indicate that employing different BDG cut‐off values for different types of invasive Candida infections may be of clinical relevance. Nonetheless, future studies involving larger patient cohorts are necessary to further investigate this observation.

BDG levels may also be influenced by the *Candida* species responsible for the bloodstream infection, and previous studies have shown that patients with candidemia caused by *C. parapsilosis* and *C. auris* may have lower levels of BDG in serum compared to patients with *C. albicans* [18, 19] and *C. tropicalis* [18]. In this study, however, we found no statistically significant association between the most common yeast species and positive BDG, although the number of cases is too low to exclude that there may be a true association.

The 30‐ and 90‐day mortality of 35% and 43% in this study was similar to that reported in a large European study on candidemia (38% and 43% respectively) [24], indicating that the population of this study was not significantly skewed towards more or less severely ill patients. A positive BDG test was associated with death at 90 days also when adjusting for other variables associated with mortality, such as age, septic shock, and ICU admission. There was a trend towards an association between a positive BDG test and 30‐day mortality that fell short of being statistically significant, possibly due to an insufficient sample size. An association between BDG concentration and mortality has also been found in previous studies [20, 22, 25, 26]. Again, a possible explanation could be that infections with a higher fungal load and with a longer duration are associated with both a higher serum BDG concentration and increased disease severity. In a study by White et al., patients that underwent abdominal surgery and who had a positive BDG test had a higher mortality irrespective of whether they had invasive candidiasis or not [26]. The authors propose that a mechanism behind this observation might be immune activation by BDG leaking through a damaged gut mucosa. An alternative explanation for the association of BDG positivity with mortality could be that elevated BDG concentrations are proxy markers for reduced integrity of the gut mucosa, reflecting more severe disease overall, rather than directly contributing to the increased mortality. Although there are many possible mechanisms other than a direct causality between BDG positivity and risk of death, a positive BDG may be used as a clinical marker for a poorer prognosis in patients with candidemia.

A strength in this study is that our results are based on two separate assays for BDG analysis. This reduces the risk that measuring errors specific to a single method are a concern. Although both assays are based on *Limulus* amoebocyte lysate, there are methodologic and operational differences that may cause assay‐specific variations. Another strength is that all patients in the study were proven cases of invasive candidiasis, which further increases the reliability of our results. Conversely, the retrospective design is a weakness that may have influenced the classification of the type of infections, where e.g., deep‐seated infections could go unrecognised due to inconsistent use of diagnostic procedures. Another limitation related to the retrospective design is that BDG testing was not standardised. Although the time frame of BDG sampling in this study represents a clinically relevant diagnostic window, interpretation of results would have been more straightforward if the sampling date were identical for all patients. Future prospective studies with standardised protocols of repeated BDG testing are warranted. Furthermore, although the sample size is larger than that of many studies in the field, it is still too limited to rule out the possibility that a true association between the outcome and some of the analysed variables may have gone undetected.

In conclusion, our results show that in the setting of candidemia, patients with deep‐seated infections other than IAC are more likely to have a positive BDG test, irrespective of the method of BDG analysis. Moreover, a positive BDG test appears to be associated with an increased mortality at 90 days. In a clinical context, our findings suggest that elevated BDG results in patients with candidemia may warrant investigation of a deep‐seated focus of infection and could be indicative of an unfavourable outcome. Prospective studies are warranted to confirm our findings.

## Author Contributions

**Karl Oldberg:** conceptualization, investigation, writing – original draft, methodology, visualization, writing – review and editing, formal analysis. **Jakob Stenmark:** investigation, writing – review and editing, methodology, formal analysis. **Helena Hammarström:** conceptualization, investigation, writing – original draft, methodology, visualization, writing – review and editing, formal analysis, project administration, supervision.

## Ethics Statement

The authors confirm that the ethical policies of the journal, as noted on the journal's author guidelines page, have been adhered to and the appropriate ethical review committee approval has been received.

## Conflicts of Interest

Fujifilm Wako provided reagents for the analysis with their instrument for free. The company did not take part in the design of the study or in the interpretation of the results. We report no further conflicts of interest.

## Supporting information


Tables S1–S4.

## Data Availability

The data that support the findings of this study are available from the corresponding author upon reasonable request.
