# Supplementary material for: β‐D‐Glucan Testing in Candidemia: Determinants of Positivity and Association With Mortality
Source: Mycoses. 2025 May 12;68(5):e70067. doi: 10.1111/myc.70067 (PMC12068012; doi:10.1111/myc.70067)
Supplement: Supplementary file 1 — Tables S1–S4. [file MYC-68-e70067-s001.docx]

| **Suppl Table 1** Univariable and multivariable analyses of mortality at 30 days, with positive Glucatell BDG-test as primary predictor, in patients with candidemia. Note that possible collinearity between other variables than BDG has not been excluded, these variables are only included for adjusting the estimate of BDG. | | | | | | | | |
| --- | --- | --- | --- | --- | --- | --- | --- | --- |
| Variable | | | Alive 30 d (n=87) | Dead 30 d (n=47) | Univariate OR  (95% CI) | P univariate | Adjusted OR (95% CI) | P multivariable |
| **Glucatell BDG-test positive** | | | **57 (66%)** | **38 (81%)** | **2.22 (0.978 – 5.44)** | **0.066** | **2.25 (0.898 – 6.06)** | **0.093** |
| Age < 18 years (n = 13) | | | 12 (14%) | 1 (2%) | 0.136 (0.00734 – 0.724) | 0.059 | 0.176 (0.00833 – 1.21) | 0.14 |
| Male | | |  |  |  |  |  |  |
| ICU admission (n = 67) | | | 33 (38%) | 34 (72%) | 4.28 (2.02 – 9.52) | <0.001 | 2.46 (0.960 – 6.46) | 0.063 |
| Species in blood culture^#^ | | |  |  |  |  |  |  |
|  | C. albicans (n = 66) | | 44 (51%) | 22 (47%) | 0.886 (0.425 – 1.85) | 0.75 |  |  |
|  | C. glabrata (n = 20) | | 12 (14%) | 8 (17%) | 1.31 (0.477 – 3.47) | 0.58 |  |  |
|  | C. parapsilosis (n = 12) | | 9 (10%) | 3 (6%) | 0.602 (0.128 – 2.15) | 0.46 |  |  |
|  | Other species (n = 29) | | 18 (21%) | 11 (23%) | 1.20 (0.499 – 2.82) | 0.67 |  |  |
| Haematologic malignancy ( n= 18) | | | 15 (17%) | 3 (6%) | 0.327 (0.0728 – 1.06) | 0.091 | 0.579 (0.120 – 2.13) | 0.44 |
| Neutropenia* (n = 24) | | | 18 (21%) | 6 (13%) | 0.553 (0.188 – 1.44) | 0.25 |  |  |
| Abdominal surgery (n = 38) | | | 24 (28%) | 14 (30%) | 1.11 (0.502 – 2.42) | 0.79 |  |  |
| Other surgery (n = 21) | | | 11 (13%) | 10 (21%) | 1.87 (0.718 – 4.82) | 0.19 | 1.11 (0.371 – 3.27) | 0.85 |
| Septic shock (n = 23) | | | 7 (8%) | 16 (34%) | 5.90 (2.29 – 16.7) | <0.001 | 3.81 (1.29 – 12.4) | 0.019 |
| Type of infection† | | |  |  |  |  |  |  |
|  | Deep seated infection (n = 38) | | 24 (28%) | 14 (30%) | 1.11 (0.502 – 2.42) | 0.79 |  |  |
|  | | IAC (n = 21) | 15 (17%) | 6 (13%) | 0.764 (0.253 – 2.07) | 0.61 |  |  |
|  | | Other deep-seated infection (n = 17) | 9 (10%) | 8 (17%) | 1.70 (0.587 – 4.85) | 0.32 |  |  |
| *BDG,* beta-D-glucan. *ICU,* intensive care unit. *IAC,* intra-abdominal candidiasis.  Categorical variables are presented with number and proportion (column percentage). Odds ratios (OR) are presented with 95% confidence intervals (95% CI).  † IAC and other deep seated infection are subgroups. Both subgroups were included in multivariable analysis, candidemia only is the reference category.  ^#^ Seven cases with mixed species were excluded.* Missing data: Neutropenia n= 1, TTP n = 1. | | | | | | | | |

| **Suppl Table 2** Univariable and multivariable analyses of mortality at 30 days, with positive Wako BDG-test as primary predictor, in patients with candidemia. Note that possible collinearity between other variables than BDG has not been excluded, these variables are only included for adjusting the estimate of BDG. | | | | | | | | |
| --- | --- | --- | --- | --- | --- | --- | --- | --- |
| Variable | | | Alive 30 d (n=87) | Dead 30 d (n=47) | Univariate OR  (95% CI) | P univariate | Adjusted OR (95% CI) | P multivariable |
| **Wako BDG-test positive** | | | **56 (64%)** | **38 (81%)** | **2.34 (1.03 – 5.72)** | **0.050** | **2.35 (0.942 – 6.27)** | **0.076** |
| Age < 18 years (n = 13) | | | 12 (14%) | 1 (2%) | 0.136 (0.00734 – 0.724) | 0.059 | 0.173 (0.00814 – 1.19) | 0.13 |
| Male | | |  |  |  |  |  |  |
| ICU admission (n = 67) | | | 33 (38%) | 34 (72%) | 4.28 (2.02 – 9.52) | <0.001 | 2.41 (0.941 – 6.34) | 0.069 |
| Species in blood culture^#^ | | |  |  |  |  |  |  |
|  | C. albicans (n = 66) | | 44 (51%) | 22 (47%) | 0.886 (0.425 – 1.85) | 0.75 |  |  |
|  | C. glabrata (n = 20) | | 12 (14%) | 8 (17%) | 1.31 (0.477 – 3.47) | 0.58 |  |  |
|  | C. parapsilosis (n = 12) | | 9 (10%) | 3 (6%) | 0.602 (0.128 – 2.15) | 0.46 |  |  |
|  | Other species (n = 29) | | 18 (21%) | 11 (23%) | 1.20 (0.499 – 2.82) | 0.67 |  |  |
| Haematologic malignancy ( n= 18) | | | 15 (17%) | 3 (6%) | 0.327 (0.0728 – 1.06) | 0.091 | 0.573 (0.119 – 2.11) | 0.43 |
| Neutropenia* (n = 24) | | | 18 (21%) | 6 (13%) | 0.553 (0.188 – 1.44) | 0.25 |  |  |
| Abdominal surgery (n = 38) | | | 24 (28%) | 14 (30%) | 1.11 (0.502 – 2.42) | 0.79 |  |  |
| Other surgery (n = 21) | | | 11 (13%) | 10 (21%) | 1.87 (0.718 – 4.82) | 0.19 | 1.15 (0.382 – 3.45) | 0.80 |
| Septic shock (n = 23) | | | 7 (8%) | 16 (34%) | 5.90 (2.29 – 16.7) | <0.001 | 3.81 (1.29 – 12.4) | 0.019 |
| Type of infection† | | |  |  |  |  |  |  |
|  | Deep seated infection (n = 38) | | 24 (28%) | 14 (30%) | 1.11 (0.502 – 2.42) | 0.79 |  |  |
|  | | IAC (n = 21) | 15 (17%) | 6 (13%) | 0.764 (0.253 – 2.07) | 0.61 |  |  |
|  | | Other deep-seated infection (n = 17) | 9 (10%) | 8 (17%) | 1.70 (0.587 – 4.85) | 0.32 |  |  |
| *BDG,* beta-D-glucan. *ICU,* intensive care unit. *IAC,* intra-abdominal candidiasis.  Categorical variables are presented with number and proportion (column percentage). Odds ratios (OR) are presented with 95% confidence intervals (95% CI).  † IAC and other deep seated infection are subgroups. Both subgroups were included in multivariable analysis, candidemia only is the reference category.  ^#^ Seven cases with mixed species were excluded.* Missing data: Neutropenia n= 1, TTP n = 1. | | | | | | | | |

| **Suppl Table 3** Univariable and multivariable analyses of mortality at 90 days, with positive Glucatell BDG-test as primary predictor, in patients with candidemia. Note that possible collinearity between other variables than BDG has not been excluded, these variables are only included for adjusting the estimate of BDG. | | | | | | | | |
| --- | --- | --- | --- | --- | --- | --- | --- | --- |
| Variable | | | Alive 90 d (n=76) | Dead 90 d (n=58) | Univariate OR  (95% CI) | P univariate | Adjusted OR (95% CI) | P multivariable |
| **Glucatell BDG-test positive** | | | **47 (62%)** | **48 (83%)** | **2.96 (1.33 – 7.02)** | **0.0098** | **3.59 (1.33 – 10.6)** | **0.015** |
| Age < 18 years (n = 13) | | | 11 (15%) | 2 (3%) | 0.211 (0.0318–0.829) | 0.049 | 0.162 (0.0129 – 1.12) | 0.10 |
| Male | | | 48 (63%) | 41 (71%) | 1.41 (0.680 – 2.96) | 0.36 |  |  |
| ICU admission (n = 67) | | | 26 (34%) | 41 (71%) | 4.63 (2.25 – 9.90) | < 0.001 | 5.13 (1.83 – 15.6) | 0.0026 |
| Species in blood culture^#^ | | |  |  |  |  |  |  |
|  | C. albicans (n = 66) | | 38 (50%) | 28 (48%) | 0.992 (0.490 - 2.01) | 0.98 |  |  |
|  | C. glabrata (n = 20) | | 12 (16%) | 8 (14%) | 0.884 (0.323 - 2.31) | 0.80 |  |  |
|  | C. parapsilosis (n = 12) | | 9 (12%) | 3 (5%) | 0.418 (0.0893 - 1.48) | 0.21 |  |  |
|  | Other species (n = 29) | | 14 (18%) | 15 (26%) | 1.62 (0.703 - 3.76) | 0.26 |  |  |
| Haematologic malignancy ( n= 18) | | | 13 (17%) | 5 (9%) | 0.457 (0.139 – 1.30) | 0.16 | 0.904 (0.226 – 3.20) | 0.88 |
| Neutropenia* (n = 24) | | | 15 (20%) | 9 (16%) | 0.735 (0.286 – 1.80) | 0.51 |  |  |
| Abdominal surgery (n = 38) | | | 21 (28%) | 17 (29%) | 1.09 (0.506 – 2.31) | 0.83 |  |  |
| Other surgery (n = 21) | | | 9 (12%) | 12 (21%) | 1.94 (0.761 – 5.12) | 0.17 | 0.628 (0.185 – 2.08) | 0.45 |
| Septic shock (n = 23) | | | 4 (5%) | 19 (33%) | 8.77 (3.05 – 32.9) | < 0.001 | 11.7 (2.85 – 68.7) | 0.0020 |
| Type of infection† | | |  |  |  |  |  |  |
|  | Deep seated infection (n = 38) | | 20 (26%) | 18 (31%) | 1.26 (0.589 – 2.69) | 0.55 |  |  |
|  | | IAC (n = 21) | 13 (17%) | 8 (14%) | 0.862 (0.315 – 2.24) | 0.76 | 0.129 (0.0230 – 0.528) | 0.0087 |
|  | | Other deep-seated infection (n = 17) | 7 (9%) | 10 (17%) | 2.00 (0.708 – 5.94) | 0.19 | 2.51 (0.699 – 9.62) | 0.16 |
| *BDG,* beta-D-glucan. *ICU,* intensive care unit. *IAC,* intra-abdominal candidiasis  Categorical variables are presented with number and proportion (column percentage). Odds ratios (OR) are presented with 95% confidence intervals (95% CI).  † IAC and other deep seated infection are subgroups. Both subgroups were included in multivariable analysis, candidemia only is the reference category.  ^#^ Seven cases with mixed species were excluded.* Missing data: Neutropenia n= 1, TTP n = 1. | | | | | | | | |

| **Suppl Table 4** Univariable and multivariable analyses of mortality at 90 days, with positive Wako BDG-test as primary predictor, in patients with candidemia. Note that possible collinearity between other variables than BDG has not been excluded, these variables are only included for adjusting the estimate of BDG. | | | | | | | | |
| --- | --- | --- | --- | --- | --- | --- | --- | --- |
| Variable | | | Alive 90 d (n=76) | Dead 90 d (n=58) | Univariate OR  (95% CI) | P univariate | AdjustedOR (95% CI) | P multivariable |
| **Wako BDG-test positive** | | | **45 (59%)** | **49 (85%)** | 3.75 (1.66 – 9.16) | 0.0022 | **4.73 (1.71 – 14.7)** | **0.0043** |
| Age < 18 years (n = 13) | | | 11 (15%) | 2 (3%) | 0.211 (0.0318–0.829) | 0.049 | 0.163 (0.0125 – 1.17) | 0.11 |
| Male | | | 48 (63%) | 41 (71%) | 1.41 (0.680 – 2.96) | 0.36 |  |  |
| ICU admission (n = 67) | | | 26 (34%) | 41 (71%) | 4.63 (2.25 – 9.90) | < 0.001 | 5.45 (1.89 – 17.1) | 0.0024 |
| Species in blood culture^#^ | | |  |  |  |  |  |  |
|  | C. albicans (n = 66) | | 38 (50%) | 28 (48%) | 0.992 (0.490 - 2.01) | 0.98 |  |  |
|  | C. glabrata (n = 20) | | 12 (16%) | 8 (14%) | 0.884 (0.323 - 2.31) | 0.80 |  |  |
|  | C. parapsilosis (n = 12) | | 9 (12%) | 3 (5%) | 0.418 (0.0893 - 1.48) | 0.21 |  |  |
|  | Other species (n = 29) | | 14 (18%) | 15 (26%) | 1.62 (0.703 - 3.76) | 0.26 |  |  |
| Haematologic malignancy ( n= 18) | | | 13 (17%) | 5 (9%) | 0.457 (0.139 – 1.30) | 0.16 | 0.924 (0.230 – 3.30) | 0.91 |
| Neutropenia* (n = 24) | | | 15 (20%) | 9 (16%) | 0.735 (0.286 – 1.80) | 0.51 |  |  |
| Abdominal surgery (n = 38) | | | 21 (28%) | 17 (29%) | 1.09 (0.506 – 2.31) | 0.83 |  |  |
| Other surgery (n = 21) | | | 9 (12%) | 12 (21%) | 1.94 (0.761 – 5.12) | 0.17 | 0.668 (0.188 – 2.34) | 0.53 |
| Septic shock (n = 23) | | | 4 (5%) | 19 (33%) | 8.77 (3.05 – 32.9) | < 0.001 | 11.6 (2.78 – 68.5) | 0.0022 |
| Type of infection† | | |  |  |  |  |  |  |
|  | Deep seated infection (n = 38) | | 20 (26%) | 18 (31%) | 1.26 (0.589 – 2.69) | 0.55 |  |  |
|  | | IAC (n = 21) | 13 (17%) | 8 (14%) | 0.862 (0.315 – 2.24) | 0.76 | 0.125 (0.0219 – 0.524) | 0.0086 |
|  | | Other deep-seated infection (n = 17) | 7 (9%) | 10 (17%) | 2.00 (0.708 – 5.94) | 0.19 | 2.33 (0.640 – 9.05) | 0.21 |
| *BDG,* beta-D-glucan. *ICU,* intensive care unit. *IAC,* intra-abdominal candidiasis  Categorical variables are presented with number and proportion (column percentage). Odds ratios (OR) are presented with 95% confidence intervals (95% CI).  † IAC and other deep seated infection are subgroups. Both subgroups were included in multivariable analysis, candidemia only is the reference category.  ^#^ Seven cases with mixed species were excluded.* Missing data: Neutropenia n= 1, TTP n = 1. | | | | | | | | |
